# The microbiome as a therapeutic co-driver in melanoma immuno-oncology

**DOI:** 10.3389/fmed.2025.1673880

**Published:** 2025-09-15

**Authors:** Jhommara Bautista, Jaime Andrés Villegas-Chávez, Doménica Bunces-Larco, Rafael Martín-Aguilera, Andrés López-Cortés

**Affiliations:** ^1^Cancer Research Group (CRG), Faculty of Medicine, Universidad de Las Américas, Quito, Ecuador; ^2^Facultade de Ciencias, Campus de A Zapateira, Universidade da Coruña, A Coruña, Spain; ^3^Esticca Medical Center, Medicina Estética Especializada, Quito, Ecuador; ^4^Department of Chemical Engineering, Universidad de Granada, Granada, Spain

**Keywords:** melanoma, immune checkpoint inhibitors, microbiome, dysbiosis, *Akkermansia muciniphila*, *Faecalibacterium prausnitzii*, antigen presentation, effector T cell activity

## Abstract

Melanoma, one of the most aggressive skin cancers, remains a major clinical challenge due to its high metastatic potential, therapy resistance, and rising global incidence. Although immune checkpoint inhibitors have transformed management, variable responses and acquired resistance limit durable benefit. Emerging evidence positions the microbiome as a pivotal determinant of melanoma biology and therapeutic outcomes. Dysbiosis in the skin, gut, and oral compartments fosters tumor-promoting inflammation, immune evasion, and oncogenic signaling, whereas enrichment of specific commensals, such as *Akkermansia muciniphila* and *Faecalibacterium prausnitzii*, enhances antigen presentation and effector T cell activity, improving ICI efficacy. Mechanistically, microbial metabolites, including short-chain fatty acids, tryptophan derivatives, and bile acids, modulate epigenetic programs, G-protein–coupled receptor signaling, and oncogenic cascades such as PI3K–AKT and RAS–RAF–MEK–ERK. Beyond the gut, cutaneous microbiota such as *Staphylococcus epidermidis* exert direct antitumor effects, while pathogenic oral taxa propagate systemic inflammation that shapes the melanoma tumor microenvironment. These insights are driving the development of microbiome-targeted interventions, including fecal microbiota transplantation, defined consortia, probiotics, and dietary modulation, with early clinical studies showing the potential to overcome resistance to immunotherapy. Integration of circadian biology further suggests that host–microbiome–immune interactions are temporally regulated, opening new dimensions for therapeutic optimization. By synthesizing mechanistic, clinical, and translational advances, this review highlights the microbiome as both a biomarker and a therapeutic axis in melanoma, underscoring its promise to transform precision immuno-oncology.

## Introduction

Melanoma is a malignancy originating from melanocytes, pigment-producing cells derived from neural crest stem cells. While these cells primarily localize to the epidermis, they also migrate to extra-cutaneous sites such as the eyes, mucous membranes, meninges, and esophagus. Accordingly, melanoma is classified into three major subtypes: cutaneous melanoma, the most common, mucosal melanoma, and uveal melanoma, reflecting the site of origin of the transformed melanocytes (1). Cutaneous melanoma is among the most aggressive malignant skin neoplasms, noted for its high metastatic potential and resistance to therapy. According to GLOBOCAN 2022, approximately 331,647 new cases and 58,645 melanoma-related deaths were reported globally, accounting for roughly 1.7% of all cancer deaths. Incidence of melanoma was highest in Australia and New Zealand, with age-standardized rates reaching 37 per 100,000 in Australia and 29.8 per 100,000 in New Zealand, followed closely by Northern and Western Europe, particularly countries like Denmark, Norway, and the Netherlands (25–31 per 100,000). North America also showed elevated incidence, with rates of 16.5 per 100,000 in the United States and 14.5 per 100,000 in Canada. In stark contrast, incidence remained low across most Asian and African countries, typically below 1 per 100,000, except for isolated areas like Israel and Namibia. Mortality patterns were more moderate but still peaked in New Zealand (3.9 per 100,000), followed by Northern Europe and Oceania, while remaining very low in Asia and Africa (< 0.5 per 100,000 in most countries) (2). Projections suggest a substantial rise by 2040, with an estimated 510,000 cases and 96,000 deaths, reflecting a ∼50% increase in incidence and a ∼68% increase in mortality if current trends persist (3).

The immune system plays a fundamental role in melanoma surveillance and progression. High infiltration of cytotoxic CD8^+^ T lymphocytes within the tumor microenvironment (TME) correlates with favorable outcomes (4). However, melanoma cells frequently evade immune detection through mechanisms such as PD-L1 overexpression, which inhibits T cell activation (5). This understanding catalyzed the development of immune ICIs, including ipilimumab (anti-CTLA-4) and pembrolizumab (anti-PD-1), which have dramatically improved survival outcomes for patients with advanced disease (6, 7). Nonetheless, variable patient responses and the emergence of primary or acquired resistance remain major therapeutic barriers. As a result, combination strategies are being explored to enhance ICI efficacy and mitigate resistance, incorporating additional therapeutic modalities (8).

Recent advances have highlighted the intestinal microbiome as a pivotal modulator of host immunity and a critical determinant of cancer immunotherapy outcomes, particularly in melanoma (9). Microbial profiles enriched in beneficial commensals such as *Akkermansia muciniphila* are associated with improved responses to ICIs, while dysbiosis has been linked to reduced therapeutic efficacy. Beyond systemic effects, tumor-resident microbial communities can also shape the local immune microenvironment by promoting cytotoxic T cell infiltration and modulating cytokine signaling pathways, ultimately enhancing antitumor immunity (10).

These insights have catalyzed the development of microbiome-targeted interventions, including fecal microbiota transplantation (FMT), which has shown promise in restoring immunotherapeutic responses in melanoma patients resistant to ICIs (8, 11). As the microbiome is increasingly recognized as both a predictive biomarker and a therapeutic target, strategies such as dietary modulation are relevant. Notably, high-fiber diets have been demonstrated to enrich immunostimulatory microbial taxa and improve ICI efficacy in both preclinical and clinical settings (12). This growing body of evidence marks a paradigm shift in immuno-oncology by positioning the microbiome as an integral determinant of treatment outcomes.

Recent analyses have proposed that gut microbiota influences melanoma through three interconnected routes: immune regulation, aging-associated changes, and modulation of the endocrine system, each contributing to tumor initiation, progression, and treatment response. This multidimensional framework highlights the need to integrate systemic host factors when assessing microbiome–melanoma interactions (13).

The objective of this review is to examine the multifaceted interplay between the microbiome and melanoma across three interrelated dimensions: (a) molecular mechanisms through which microbial communities promote or suppress tumorigenesis; (b) immune modulation, with emphasis on how the microbiota shapes innate and adaptive immune responses and influences tumor immunogenicity; and (c) therapeutic strategies, including emerging microbiome-based interventions such as FMT and dietary modification as adjuncts to immunotherapy. By synthesizing findings from recent high-impact studies, this review aims to provide a comprehensive and updated perspective to guide clinical decision-making and inform future directions in melanoma immuno-oncology.

## Characterization of the microbiome in the context of melanoma

### Skin microbiome in melanoma

**FIGURE 1:**
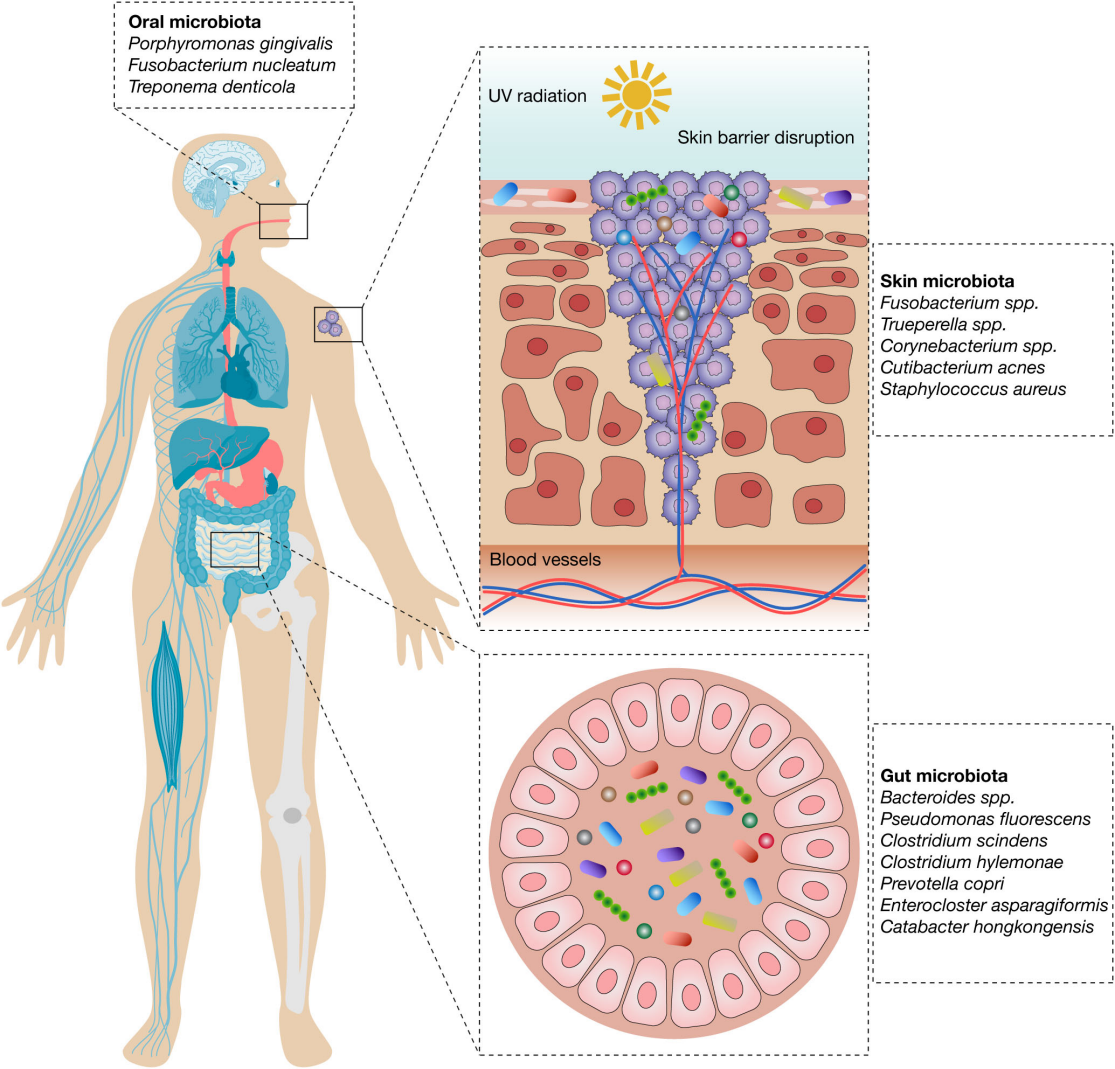
Microbial signatures of the skin, gut, and oral microbiomes associated with melanoma progression and/or dysbiosis. The figure depicts representative bacterial taxa for each compartment linked to tumor-promoting inflammation, immune evasion, or microenvironmental changes that favor melanoma development.

The human skin microbiome is a dynamic and site-specific ecosystem dominated by Actinomycetota, Bacillota, Pseudomonadota and Bacteroidota, which maintain epidermal homeostasis, immune protection, and barrier integrity (14, 15). In healthy individuals, taxonomic composition varies depending on moisture, sebaceous activity and environmental exposure, contributing to a balanced cutaneous immune microenvironment. In melanoma-affected skin, however, 16S rRNA sequencing analyses reveal significant dysbiosis, characterized by reduced microbial diversity and the enrichment of proinflammatory taxa such as *Fusobacterium* and *Trueperella* (4) (Figure 1). These alterations are associated with the establishment of tumor-promoting inflammatory signaling pathways. Mendelian randomization analyses have also uncovered bidirectional causal relationships between specific microbial classes (e.g., Bacilli and Betaproteobacteria) and melanoma risk (5), further underscoring the etiological relevance of skin microbial composition. Conversely, beneficial commensals such as *Staphylococcus epidermidis* exert antitumor activity through the production of 6-N-hydroxyaminopurine (6-HAP), a selective DNA synthesis inhibitor, and via the modulation of local IL-17 signaling pathways (16). Other commensals, including *Corynebacterium* spp., contribute to immune homeostasis, whereas typical skin residents such as *Cutibacterium acnes* and *Staphylococcus aureus* can, under dysbiotic conditions, drive inflammatory responses that favor immune evasion and disrupt epithelial integrity (17). Functional pathway analyses show that metabolic and immune-related microbial activities are significantly altered in melanoma lesions, suggesting a mechanistic link between cutaneous dysbiosis and tumor progression (4, 6).

### Gut microbiome in melanoma

The gut microbiota plays a central role in immune regulation and has emerged as a critical factor influencing both melanoma pathophysiology and the efficacy of immunotherapies, particularly ICIs, in the treatment of melanoma (18, 19). One of the primary mechanisms by which the gut microbiota exerts its influence is through the production of microbial metabolites, particularly short-chain fatty acids (SCFAs), such as butyrate, propionate and acetate (19). These SCFAs are synthesized by beneficial commensals, including *Faecalibacterium prausnitzii* and *Roseburia* spp., and mediate immunoregulatory functions by activating G-protein-coupled receptors (GPR41, GPR43, GPR109A) and inhibiting histone deacetylases (HDACs) (19, 20). Favorable responses to ICIs have been associated with enrichment of specific bacterial taxa, including *A. muciniphila* and *F. prausnitzii*, which enhance dendritic cell activation and effector T-cell responses. In contrast, an overrepresentation of *Bacteroidota* correlates with impaired immunotherapy outcomes (21). FMT from ICI responders into non-responders has demonstrated improved clinical responses and increased intratumoral T-cell infiltration (10, 11). Moreover, longitudinal analyses highlight that gut microbial composition evolves during therapy, supporting its potential use in dynamic monitoring to guide treatment decisions. Nevertheless, the interaction between gut microbiota and ICI outcomes is highly heterogeneous. Studies report both beneficial and detrimental associations for the same taxa, reflecting variability arising from host factors (e.g., genetics, diet, prior therapies) as well as methodological differences in microbiome profiling. Importantly, strain-level resolution is increasingly recognized as critical, since bacterial strains belonging to the same species can exert divergent immunomodulatory effects. Gunjur et al. (22) demonstrated that strain-based models better predict ICI outcomes, underscoring the need for precision microbiome analyses in immuno-oncology (22).

Mechanistically, SCFAs such as acetate, propionate, and butyrate bind to cell-surface G-protein–coupled receptors (GPR41/FFAR3, GPR43/FFAR2, and GPR109A/HCAR2) expressed on epithelial cells, dendritic cells, macrophages, and subsets of T cells. Activation of GPR41 and GPR43 triggers downstream ERK1/2, p38 MAPK, and PI3K–AKT signaling, leading to increased production of anti-inflammatory cytokines (e.g., IL-10) and enhanced chemotaxis of immune cells to the TME (23, 24). GPR109A, preferentially activated by butyrate, promotes differentiation of regulatory T cells (Tregs) and IL-10–producing macrophages, contributing to immune homeostasis (25).

In parallel, SCFAs enter immune and tumor cells via monocarboxylate transporters (MCT1/4) and directly inhibit class I and IIa histone deacetylases (HDACs). This epigenetic regulation increases histone acetylation at promoters of genes involved in cell cycle arrest (e.g., *p21*), apoptosis, and immune activation (e.g., *IFNG*, *IL12B*) (26, 27). In melanoma models, butyrate-mediated HDAC inhibition has been shown to enhance MHC class I and II expression on tumor cells, improving antigen presentation and responsiveness to checkpoint blockade (28). The balance between GPR-mediated anti-inflammatory signaling and HDAC-driven transcriptional reprogramming is therefore critical; low to moderate SCFA levels may enhance antitumor immunity, whereas chronically elevated concentrations can skew the immune environment toward tolerance and Treg dominance. However, while SCFAs support immune homeostasis, persistently elevated levels may paradoxically foster an overly immunosuppressive microenvironment. This environment is often marked by an excessive presence of Tregs, facilitating immune evasion by tumor cells and reducing the therapeutic efficacy of immune checkpoint inhibitors (ICIs), particularly those targeting the PD-1/PD-L1 axis (29).

Beyond SCFAs, other microbiota-derived metabolites relevant to melanoma immunity include kynurenine (KYNA), urolithin A (UA), and lithocholic acid (LCA), each derived from distinct dietary or host substrates. KYNA originates from dietary tryptophan via the indoleamine 2,3-dioxygenase (IDO) and tryptophan 2,3-dioxygenase (TDO) pathways, which can be activated by both host immune cells and gut microbes such as *Pseudomonas fluorescens* and *Lactobacillus spp.* KYNA acts via the aryl hydrocarbon receptor (AhR) to promote Treg expansion and suppress cytotoxic T cell activity, contributing to immune escape in melanoma (30, 31). UA is generated from ellagitannins and ellagic acid, abundant in pomegranates, berries, and nuts, by *Gordonibacter urolithinfaciens* and *Ellagibacter spp.* UA has been shown to attenuate NF-κB signaling, enhance mitochondrial function in immune cells, and support Th1-polarized responses, thereby reducing tumor-promoting inflammation (32, 33). LCA, a secondary bile acid, is produced through the 7α-dehydroxylation of primary bile acids by *Clostridium scindens* and *Clostridium hylemonae*. In melanoma, LCA can activate ERK1/2 signaling, upregulate c-Myc and Cyclin D, and promote tumor cell proliferation, while also impairing dendritic cell maturation and skewing macrophage polarization toward immunosuppressive M2 phenotypes (33, 34).

Emerging evidence indicates that gut microbiota composition differs between melanoma stages, with progressive disease often showing reduced microbial diversity and enrichment of specific taxa such as *Bacteroides*. Certain strains, including *Lactobacillus reuteri* FLRE5K1 and *Bifidobacterium longum* BB-536, have demonstrated antitumor effects in murine melanoma models by modulating cytokine production, enhancing CD8^+^ T cell infiltration, and suppressing pro-tumor inflammatory mediators (13). Together, these findings support the role of the gut microbiome both as a predictive biomarker and as a co-target in melanoma immunotherapy (Figure 1).

### Oral microbiome in melanoma

The oral microbiome consists of more than 700 microbial species and plays a central role in maintaining systemic immune homeostasis. Dysbiosis of the oral microbiota has been implicated in several inflammatory diseases, and evidence suggests it may indirectly impact melanoma progression (12). Pathogenic taxa such as *Porphyromonas gingivalis*, *Fusobacterium nucleatum*, and *Treponema denticola* (the “red complex”) can enter the circulation during mastication or in the context of periodontal inflammation. Their structural components, including lipopolysaccharide and gingipains, activate TLR2 and TLR4 signaling in immune and endothelial cells, resulting in NF-κB activation and secretion of proinflammatory cytokines such as IL-6, TNF-α, and CCL2 (35, 36) (Figure 1).

Recent evidence has begun to elucidate the mechanisms linking oral dysbiosis to melanoma biology through systemic inflammatory pathways. Pathogenic taxa such as *P. gingivalis*, *F. nucleatum*, and *T. denticola*, hallmark members of the periodontitis-associated “red complex,” can enter the bloodstream during routine mastication or in the presence of periodontal inflammation (12). Their structural components, including lipopolysaccharide (LPS) and gingipains, activate Toll-like receptor 2 (TLR2) and Toll-like receptor 4 (TLR4) on monocytes, macrophages, and endothelial cells, triggering NF-κB signaling and promoting secretion of proinflammatory cytokines such as IL-6, TNF-α, and CCL2 (35, 36). These mediators drive chronic systemic inflammation, which can facilitate melanoma immune evasion via M2 macrophage polarization, expansion of myeloid-derived suppressor cells (MDSCs), and induction of T cell exhaustion (37). In addition, oral pathobionts produce metabolites, such as polyamines, hydrogen sulfide, and altered arginine derivatives, that modulate T cell and natural killer (NK) cell activity, potentially reshaping the cutaneous TME (12).

The influence of the oral microbiome on melanoma may also occur indirectly through the gut–skin axis. Swallowed oral bacteria can transiently colonize or alter gut microbial communities, leading to changes in SCFA and bile acid profiles that regulate systemic and skin-resident immune responses (38). Gut dysbiosis resulting from oral microbial perturbation can impair cutaneous barrier integrity, promote IL-17–driven inflammation, and create a microenvironment favorable for melanocyte transformation. Conversely, interventions that restore oral–gut microbial balance, such as improved oral hygiene, targeted probiotics, or selective antimicrobial approaches, may reduce systemic inflammation and synergize with microbiome-modulating strategies already being tested to enhance melanoma immunotherapy (36, 38). Taken together, these findings support the concept of an interconnected oral-gut-skin microbial network in which dysbiosis in one compartment can propagate inflammatory and immunosuppressive signals to distal sites, ultimately impacting melanoma initiation, progression, and therapeutic responsiveness.

### Factors affecting microbiome composition

The composition and diversity of both skin and gut microbiota are shaped by multiple extrinsic factors. For the gut, diet is the most powerful modulator—high-fiber, plant-rich diets enrich SCFA-producing taxa such as *F. prausnitzii* and *A. muciniphila*, which correlate with improved responses to ICIs (39). In contrast, Western dietary patterns high in saturated fats and refined sugars promote dysbiosis, gut permeability, and systemic inflammation (40). The skin microbiota is strongly influenced by ultraviolet (UV) radiation, which can impair barrier integrity, induce local immune suppression, and shift communities toward UV-resistant taxa (41). Climate, humidity, hygiene practices, cosmetic products, and environmental exposures such as soil or animal contact further modulate skin microbial composition.

Across both compartments, pharmacologic exposures, including broad-spectrum antibiotics, proton pump inhibitors, and certain chemotherapies, can profoundly disrupt microbial balance, deplete beneficial taxa, and reduce immunotherapy efficacy while increasing the risk of immune-related adverse events (42). Immunotherapy itself can alter microbial communities, creating a bidirectional relationship in which treatment outcomes and microbiome composition are interdependent (8). Additional modulators include psychosocial stress, sleep disruption, and circadian rhythm misalignment, which can alter gut permeability and immune tone, indirectly affecting skin immunity via the gut–skin axis. Understanding these influences is essential for designing microbiome-targeted interventions to optimize melanoma immuno-oncology.

### Gut dysbiosis and oncogenic signaling in melanoma

Gut microbial dysbiosis has emerged as a recognized hallmark of cancer, due to its broad impact on key tumor-associated processes such as chronic inflammation, immune evasion, and genomic instability, all of which contribute to malignant transformation and tumor progression (43). Beyond immune modulation, the gut microbiome also impacts melanoma through the regulation of key oncogenic pathways. Microbiota-derived metabolites, including SCFAs, urolithin A (UA), and kynurenine (KYNA), modulate intracellular signaling cascades implicated in cancer progression. In particular, AKT and ERK function as central nodes in melanoma signaling networks: AKT primarily governs cell survival, metabolism, angiogenesis, and apoptosis resistance via the PI3K/AKT/mTOR axis (44, 45), while ERK, as part of the RAS–RAF–MEK–ERK cascade, drives cell cycle progression, differentiation, migration, and transcriptional activation of oncogenes such as c-Myc and regulators like Cyclin D (46).

For example, SCFAs such as butyrate and propionate can inhibit phosphorylation of both AKT and ERK, thereby suppressing pro-survival signaling, inducing apoptosis, and limiting tumor growth in melanoma models (47). Conversely, lithocholic acid (LCA), another microbial metabolite, enhances p-ERK1/2 signaling, stimulating c-Myc transcription and Cyclin D expression to promote uncontrolled proliferation. Likewise, LPS from Gram-negative bacteria activates TLR4, triggering the PI3K/AKT/mTOR pathway and thereby increasing melanoma cell proliferation, invasion, and metastatic potential (48).

In mucosal melanoma models, inhibition of the PI3K pathway using LY294002 significantly decreased AKT phosphorylation and cell viability. However, a partial restoration of AKT activity after 24 h suggests the presence of feedback mechanisms or the emergence of resistant subclones (49). These observations underscore the dynamic interplay between microbial metabolites and tumor signaling networks. Overall, the gut microbiota emerges as a critical regulator of melanoma not only through its effects on immune dynamics but also via direct modulation of oncogenic signaling pathways. By mapping how specific microbial metabolites influence AKT and ERK activation states, new microbiota-targeted or combinatorial therapies could be designed to selectively suppress tumor-promoting pathways while enhancing antitumor immunity (50).

### Influence of the skin microbiome on the malignant transformation of melanocytes

The skin microbiome plays a central role in maintaining cutaneous immune equilibrium, but its dysregulation can contribute to the malignant transformation of melanocytes. Importantly, skin microbiota interact with host immune cells not only during barrier disruption but also under homeostatic conditions. Commensals such as *Staphylococcus epidermidis* modulate keratinocyte antimicrobial peptide production, regulate inflammatory signaling, and promote regulatory T cell expansion within hair follicles, thereby sustaining immune tolerance and cutaneous homeostasis (51). One of the key mechanisms is the induction of chronic inflammation, a recognized driver of carcinogenesis that promotes uncontrolled cell proliferation and resistance to apoptosis-hallmarks of tumor development (52). This inflammatory response can be triggered by the release of microbial products such as antimicrobial peptides, LPS, and other immunomodulatory compounds from an altered skin microbiome (19). Certain bacterial species, like *Corynebacterium*, implicated in acne, can stimulate local immune activation through IL-17 signaling pathways, which are known to sustain chronic inflammation and promote carcinogenesis (53). These pathways contribute to melanoma progression by enhancing angiogenesis and cell proliferation.

Skin microorganisms can contact and modulate immune cells directly when the epidermal barrier is disrupted, such as after UV-induced injury. Ultraviolet radiation damages keratinocytes, increases permeability of the stratum corneum, and induces the release of antimicrobial peptides and alarmins (e.g., LL-37, HMGB1) that recruit and activate Langerhans cells, dermal dendritic cells, and resident T cells (41, 54). Barrier compromise facilitates translocation of commensal or opportunistic microbes, or their components such as lipoteichoic acid, peptidoglycan, and LPS, into deeper epidermal and dermal layers, where they engage pattern recognition receptors (TLRs, NOD-like receptors) on skin-resident immune cells (55, 56). This can initiate local cytokine cascades (IL-1β, TNF-α, IL-17) that shape the TME toward either immune activation or tolerance, depending on the microbial context. In melanoma-prone skin, repeated UV exposure may therefore amplify microbe–immune interactions, influencing melanocyte transformation and tumor progression.

Moreover, persistent inflammation driven by skin-resident microbes reshapes the TME, favoring infiltration by immune cells that may paradoxically support tumor growth. These cells secrete growth factors and immunosuppressive molecules that aid tumor survival rather than eradication (54). Notably, microbial profiling in melanoma patients has revealed increased abundance of *Fusobacterium* and *Trueperella*, suggesting a specific microbiome signature associated with the disease (57). In addition, several bacterial species can directly modulate Langerhans cell (LC) function. For example, *Staphylococcus aureus* produces α-toxin, which has been shown to downregulate co-stimulatory molecule expression, such as CD80 and CD86, and MHC II on antigen-presenting cells via IL-10–mediated immunomodulation (58). *Cutibacterium acnes* engages TLR2 (and potentially TLR4) on skin immune cells, including LCs, leading to production of pro-inflammatory cytokines such as IL-1β and IL-12, driving Th17 differentiation and chronic inflammation (59). LCs are known to express TLR2, making them responsive to *C. acnes* components (60). *F. nucleatum*, while more studied in colorectal cancer, is recognized as an immunomodulatory microbe that can promote IL-10 production, suppress cytotoxic immune activity, and create a tumor-promoting microenvironment (61). TLR2/TLR6 signaling, relevant for bacteria like *Trueperella* spp., can induce tolerogenic or pro-inflammatory responses depending on duration and context, potentially reprogramming LC responses toward immune tolerance (62). Collectively, these microbial interactions can impair antigen presentation by LCs, skew cytokine profiles (e.g., toward IL-10), reduce co-stimulatory signals, and foster a microenvironment favoring immune evasion and melanoma progression.

### Immune response modulated by the microbiome in melanoma

Beyond the skin, the gut microbiome has a profound influence on systemic immunity, including in the context of melanoma. It regulates the activity of innate immune cells, such as macrophages and dendritic cells, through microbial signals that engage Toll-like receptors (e.g., TLR4), initiating downstream pathways like NF-κB (19, 41).

Lipopolysaccharide, a bacterial component, activates macrophages via TLR4 to induce cytokines like IL-10 and TNF-α, helping mount an effective immune defense without triggering systemic inflammation. The microbiome is also essential for dendritic cell maturation. In germ-free (GF) mice, the absence of microbial cues prevents these cells from producing key proinflammatory cytokines (e.g., IL-6, IL-12, TNF), impairing their capacity to activate the adaptive immune system (14, 63).

Short-chain fatty acids, produced by microbial fermentation, are crucial epigenetic regulators that enhance NF-κB signaling. This promotes the expression of inflammatory cytokines like IL-1β and TNF, as well as costimulatory molecules essential for T cell and NK cell activation. Beyond SCFAs, microbial tryptophan metabolites such as kynurenine and indole-3-propionic acid can act via the AhR to modulate T-cell differentiation, while secondary bile acids influence macrophage polarization and dendritic cell function (64). Dysbiosis impairs these processes, increasing vulnerability to infections and neoplasia (65).

In melanoma, dysbiosis not only dampens cytotoxic immune responses but also favors the recruitment and expansion of immunosuppressive cell populations within the TME. These include Tregs that inhibit CD8^+^ T cells and NK cells via IL-10 and TGF-β (66, 67); MDSCs that suppress T-cell proliferation and promote angiogenesis through arginase-1, nitric oxide, and VEGF; and M2-polarized tumor-associated macrophages (TAMs) that secrete IL-10, CCL22, and growth factors supporting tumor survival (68). Additional pro-tumor infiltrates include exhausted CD8^+^ T cells expressing PD-1, LAG-3, and TIM-3; Th2-skewed CD4^+^ T cells producing IL-4 and IL-13; certain B-cell subsets that modulate the cytokine milieu; and N2-polarized neutrophils that release reactive oxygen species (ROS) and neutrophil extracellular traps (NETs) to facilitate invasion. Mechanistically, dysbiosis disrupts the regulation of innate and adaptive immunity by reducing beneficial commensal metabolites, increasing systemic exposure to pathogen-associated molecular patterns (PAMPs), and skewing the cytokine balance toward chronic inflammation and immune tolerance. Accumulating evidence indicates that gut microbiome dysbiosis directly promotes the expansion of both Tregs and MDSCs in the melanoma TME, fostering immune tolerance and resistance to immunotherapy (69, 70).

### Key bacteria in immune response modulated by the microbiome in melanoma

The gut microbiome is essential for adaptive immunity. Butyrate deficiency negatively influences the differentiation of Tregs and the activity of cytotoxic T cells (CD8+). Therefore, the microbiota enables the direct development of Tregs. SCFAs, especially acetate, can regulate the acetylation of lysine residues directly related to proteins of the adaptive immune system (71).

Thus, intestinal dysbiosis, by altering innate immunity in antigen-presenting cells, also modifies the response of the adaptive system (4). Similarly, epidermal dysbiosis alters Helper 17 T lymphocytes, with alterations in IL-17 and IL-22, possibly implicated in cancer progression (72).

Checkpoints help prevent T cells from destroying normal cells when cancer cells are present (73). ICIs block this inhibition of T cells, allowing tumor destruction. Thus, intestinal microbial enrichment of bacteria such as *Bifidobacteria* or *Bacteroides* could determine patients who respond to anti-PD-L1 therapy or checkpoint inhibitors. In studies done in murine subjects enriched with these bacteria, they obtained an improvement in tumor control and IFN-γ production (74).

Specific microbial species play outsized roles in shaping the immune landscape in melanoma. *Bacteroides fragilis* and *A. muciniphila* are among the most influential taxa (75). *B. fragilis* promotes dendritic cell maturation in both lymph nodes and the TME facilitating the activation of effector T cells while suppressing Treg-mediated immunosuppression. In melanoma models, oral administration of *B. fragilis* has been shown to restore immune function and enhance the efficacy of CTLA-4 inhibitors (76, 77). It has also been shown that colonization by *B. fragilis* or *Clostridium* stimulates the production of tumor suppressor TregFoxp3+ cells (74).

Several commensal bacterial species have demonstrated potent immunomodulatory effects that translate into superior antitumor activity in melanoma. *A. muciniphila* has shown robust tumor control *in vivo* in mice monocultured with this strain and *in vitro*, where it activates the IFN-I–NK–DC axis in the TME, resulting in a markedly stronger antitumor response compared to other microorganisms, such as *L. reuteri* (78). In addition, *A. muciniphila* promotes the infiltration of CD4^+^ CCR9^+^ CXCR3^+^ T cells into the tumor site, thereby supporting a strong Th1-skewed immune response—an essential component for effective tumor control (76). Similarly, *F. prausnitzii* has been associated with a favorable immune profile in patients receiving ICIs, characterized by an increase in CD4^+^ T cells and a reduction in Tregs, suggesting enhanced immune activation. Collectively, these data highlight the therapeutic potential of modulating the microbiome to improve immune responses and enhance the clinical efficacy of ICIs in melanoma.

## Impact of the microbiome on treatment response in melanoma

The gut microbiome has emerged as a critical modulator of therapeutic efficacy and toxicity in melanoma treatment. Its influence spans chemotherapy, immunotherapy, and radiotherapy through mechanisms such as bacterial translocation, immune modulation, metabolism, and enzymatic degradation (79). Gut microbiota composition may also influence the risk of immune-related adverse events (irAEs) during ICI therapy. For instance, high *Streptococcus* abundance has been linked to increased irAE incidence and shorter progression-free survival in melanoma patients treated with anti–PD-1, while baseline enrichment of *Bacillota* has been associated with immune-mediated colitis during ipilimumab therapy (13).

### Chemotherapy and the gut microbiome in melanoma

Studies have shown that chemotherapeutic agents like cyclophosphamide and doxorubicin can alter gut permeability, allowing translocation of commensal bacteria to secondary lymphoid organs. In murine models, this enhances helper T cell (pTh17) responses and improves chemotherapy efficacy (80). However, this beneficial immune activation comes at a cost. Disruption of the mucosal barrier can lead to inflammation and epithelial cell loss, contributing to gastrointestinal toxicity. Moreover, the use of broad-spectrum antibiotics can induce resistance to cyclophosphamide, likely by depleting beneficial microbes required for optimal therapeutic response (79). Clinical evidence further supports this link. In patients treated for non-Hodgkin lymphoma, longitudinal analysis of fecal samples revealed significant reductions in microbial diversity following chemotherapy. Although these findings have not been fully explored in melanoma, they suggest that microbiota alterations may similarly impact treatment outcomes (81).

### Immunotherapy response and microbiota influence

Immunotherapy has revolutionized melanoma treatment, particularly through ICIs targeting PD-1 and CTLA-4. However, many patients experience either primary or acquired resistance. Meta-analyses of melanoma cohorts receiving anti–PD-1 therapy have found that responders often exhibit enrichment of *Lachnospiraceae* and *Actinomycetota* (including *Bifidobacterium*), while non-responders display higher levels of *Prevotella* spp. Moreover, beneficial species such as *Coprobacillus cateniformis* and *Erysipelatoclostridium ramosum* can enhance anti–PD-L1 responses in murine models by downregulating inhibitory pathways involving PD-L2 and RGMb (13).

Microbiota-driven modulation plays a direct role in resistance phenotypes. In primary resistance, unfavorable microbiome configurations, often characterized by low diversity, depletion of SCFA-producing taxa (*F. prausnitzii*, *A. muciniphila*) and overrepresentation of immunosuppressive taxa (*B. fragilis* non-enterotoxigenic strains, *Prevotella copri*), can impair dendritic cell maturation, reduce CD8^+^ T cell priming, and promote Treg and MDSC expansion. These changes synergize with tumor-intrinsic mechanisms such as B2M mutations, MHC-I heterozygosity loss, or JAK1/2 defects to block effective immune recognition (8, 40, 77).

In acquired resistance, longitudinal studies show that microbiome composition can shift during ICI therapy toward dysbiosis, enriching taxa that promote chronic inflammation or immunosuppression, such as certain *Clostridium* cluster XIVa members. These changes are associated with reduced IFN-γ signaling, altered antigen processing, and re-establishment of an immunosuppressive TME, thereby facilitating relapse despite prior response. FMT from responders into relapsed patients has been shown to restore a favorable microbial profile, re-activate cytotoxic T cell responses, and resensitize tumors to PD-1 blockade, underscoring a causal relationship between microbial dynamics and both primary and acquired resistance (11, 82, 83).

Given these complexities, strategies that combine ICIs with microbiota-modulating interventions, such as targeted probiotics, prebiotics, diet modification, and FMT, offer a rational path to overcoming resistance and enhancing durable efficacy.

### Radiotherapy and microbiota interactions

Radiotherapy (RT) is occasionally employed in advanced melanoma, and recent findings suggest that gut microbiota composition can modulate its effectiveness. Preclinical data indicate that bacterial depletion via vancomycin enhances RT response by promoting CD8^+^ T cell infiltration and increasing intratumoral IFN-γ levels (84). In contrast, antibiotic-induced dysbiosis or tumor-mediated microbiota alterations can impair immune responses and diminish the therapeutic benefits of RT (85).

Importantly, FMT has been used to restore ICI sensitivity in melanoma patients who were previously resistant to anti-PD-1 therapy (82). This suggests that manipulating the microbiota could also potentiate the effects of RT, offering a novel strategy to improve clinical outcomes in melanoma management (80).

## Microbiome-based strategies to enhance cancer therapy

Modulating the gut microbiome has emerged as a promising strategy to improve cancer treatment outcomes, particularly when combined with immunotherapy. Interventions such as FMT, dietary modifications, and the use of prebiotics and probiotics have demonstrated the ability to enhance antitumor immune responses (80, 86).

### Probiotics and prebiotics in immune modulation

Specific probiotics, including *Lactobacillus*, *Rhamnosus*, and *Bifidobacterium longum*, promote dendritic cell maturation and CD8^+^ T cell activation, strengthening the immune response against tumor cells (87, 88). In preclinical melanoma models, administration of *Bifidobacterium bifidum* enhanced the efficacy of oxaliplatin and anti–PD-1 therapy by increasing IFN-γ^+^ CD8^+^ T cell infiltration within the TME (80). Similarly, prebiotics such as inulin, mucin, and fructooligosaccharides stimulate the production of SCFAs, like butyrate, which serve as immunological and epigenetic regulators by increasing the expression of antitumor genes.

Recent studies also show that inulin enhances ICI efficacy by promoting CD8^+^ T cell differentiation into memory stem cells (89). Furthermore, *A. muciniphila*, a beneficial gut bacterium, is associated with improved responses to PD-1 blockade in melanoma patients. Emerging studies indicate that patients with a balanced microbiota, supported by probiotics and prebiotics, show better responses to immunotherapy (40, 48). However, observational clinical data suggest that non-targeted, over-the-counter probiotics may correlate with reduced gut microbial diversity and lower response rates to ICIs in melanoma patients, highlighting the need for strain-specific, clinically tested formulations rather than indiscriminate supplementation (39, 90).

### Dietary modulation of the microbiome in melanoma

Diet is a major determinant of microbiota composition and function. High-fiber diets promote greater microbial diversity and increase the abundance of SCFA-producing bacteria such as *Faecalibacterium* and *Akkermansia*, which are linked to improved outcomes with ICIs (40, 80). A study by Spencer et al. found that melanoma patients adhering to high-fiber diets were five times more likely to respond to PD-1 blockade compared to those on low-fiber diets (39).

In contrast, Western-style diets rich in saturated fats, sugars, and processed foods promote dysbiosis, reduce SCFA production, and increase systemic inflammation. Although polyphenols have shown anti-inflammatory potential, more studies are needed to define their role in melanoma therapy. Beyond fiber, adherence to a Mediterranean diet, rich in vegetables, legumes, whole grains, fish, and olive oil, has been associated with higher objective response rates and longer progression-free survival in advanced melanoma patients on ICIs (91). Preclinical studies also suggest that ketogenic diets, or supplementation with the ketone body β-hydroxybutyrate, may enhance the efficacy of anti–PD-1 and anti–CTLA-4 therapy through T-cell–dependent mechanisms, prompting ongoing clinical trials. Large-scale nutritional trials are required to establish dietary guidelines for enhancing immunotherapy response in melanoma.

### Fecal microbiota transplantation in melanoma

Fecal microbiota transplantationhas shown promise in restoring responsiveness to immunotherapy in patients with refractory melanoma. Transplantation of fecal material from PD-1 responder donors into non-responders has reprogrammed the gut microbiota to a more immunostimulatory state, increasing therapeutic sensitivity (11).

Post-FMT analysis reveals enrichment of beneficial species such as *A. muciniphila* and *F. prausnitzii*, which correlate with increased CD8^+^ T cell activity and reduced immunosuppressive signaling (82). Furthermore, FMT enhances expression of proinflammatory cytokines and activates type I interferon signaling pathways, key elements of an effective antitumor immune response (80). These findings strongly support the clinical utility of this strategy in advanced melanoma. Nonetheless, FMT has important limitations: responses are donor-dependent and not universal; there is variability in engraftment durability; and safety concerns, including the risk of pathogen transmission (e.g., multidrug-resistant organisms), have led to strict regulatory oversight. Additionally, differences in preparation, delivery methods (capsules vs. colonoscopy), and cost of donor screening pose logistical barriers. These factors currently limit FMT’s routine use outside clinical trials, and research is focusing on standardized, defined microbial consortia as safer alternatives (92–94).

Clinical trials further support the therapeutic potential of FMT in melanoma. In PD-1–refractory patients, donor material from responders has restored antitumor immunity and improved clinical outcomes, with responder profiles enriched in *Ruminococcaceae*, *Alistipes communis*, and *Blautia* species. Conversely, non-responders often harbor *Enterocloster asparagiformis* and *Catabacter hongkongensis*, taxa associated with immunosuppressive phenotypes (13).

### Development of microbiome-targeted therapies in melanoma

Novel therapies that directly target microbiota composition are under investigation. A randomized clinical trial evaluated SER-401, an oral formulation enriched with *Bacillota*, in combination with nivolumab in patients with metastatic melanoma. Although enrollment was limited during the COVID-19 pandemic, a 25% response rate was achieved, demonstrating feasibility and safety (95, 96).

On the other hand, the use of broad-spectrum antibiotics like vancomycin has been shown to negatively affect gut microbial diversity and reduce immunotherapy efficacy, particularly in patients with high levels of *Ruminococcaceae* (96, 97). These insights underscore the need for precision microbiome modulation and the development of predictive microbial biomarkers to optimize treatment personalization.

## Conclusion and future perspectives

The global burden of cutaneous melanoma continues to rise, with projections indicating a substantial increase in incidence by 2040, emphasizing the urgent need for novel preventive and therapeutic strategies to curb its clinical impact (3). Over the past decade, significant advances have deepened our understanding of the skin microbiome as a dynamic ecosystem that contributes not only to cutaneous homeostasis but also to disease susceptibility, including oncogenesis (15). These early insights paved the way for investigations into the role of microbial dysbiosis in melanoma, revealing that imbalances in both the skin and gut microbiota can foster a microenvironment conducive to tumor progression through mechanisms involving chronic inflammation, immune evasion, and altered metabolite production (4).

Simultaneously, mounting evidence has underscored the importance of the gut microbiome in shaping host responses to cancer therapies, particularly ICIs, which have revolutionized the management of advanced melanoma. Specific microbial signatures have been associated with enhanced responsiveness to ICIs, suggesting a causal link between microbiome composition and treatment efficacy (18). Specific microbial signatures have been linked to improved ICI responsiveness, while factors such as antibiotic use can disrupt microbial homeostasis and reduce therapeutic efficacy, highlighting the microbiota as a critical player in immuno-oncology (21). In parallel, oncogenic viruses have emerged as additional modulators of cancer progression and therapy response. Beyond their well-established roles in genome integration, immune evasion, and chronic inflammation, these viruses also reshape the host epigenetic landscape-modifying DNA methylation, histone marks, and non-coding RNA expression, to promote immune escape, cellular transformation, and oncogenesis (98). This convergence of microbial and viral influences on immune regulation and epigenetic remodeling underscores the need for integrated therapeutic strategies that consider the host-microbe-epigenome axis in cancer treatment.

One of the most compelling clinical findings supporting this paradigm is the use of FMT to overcome primary resistance to anti-PD-1 therapy in patients with refractory melanoma. These interventions successfully reprogrammed the gut microbiome, restored antitumor immunity, and induced durable responses, highlighting the therapeutic potential of microbiome modulation in precision oncology (11). Such outcomes underscore a transformative concept: manipulating microbial communities may not only serve as an adjunct to current immunotherapies but could also recondition the TME to reinstate immune surveillance.

The integration of microbial research into melanoma biology is evolving from correlation to causation, as mechanistic studies now connect specific taxa or metabolites to pathways relevant for tumor immunity, angiogenesis, and metastasis (36). However, this emerging field still faces considerable challenges, including interindividual variability, host genetics, diet, environmental exposures, and the absence of standardized analytical pipelines. One promising yet underexplored dimension in this context is the role of circadian rhythms near-24 h oscillations regulating key physiological and behavioral processes such as, sleep-wake cycles, mitochondrial function, DNA repair, cellular redox, hypoxia, and immune responses, have emerged as critical modulators of both microbial dynamics and host immunity (99, 100). Future research should adopt integrative multi-omic and spatiotemporal approaches to unravel the interplay between the microbiome, circadian regulation, and melanoma progression at single-cell resolution.

Emerging concepts, such as the interaction between the skin microbiota and the local cutaneous immune system, open novel avenues for intervention. Certain commensals may modulate the activation state of skin-resident immune cells, influencing antitumor surveillance or tolerance, particularly in early melanoma or premalignant lesions (29). Moreover, microbial-derived metabolites, including SCFAs, tryptophan catabolites, and bile acid derivatives, are increasingly recognized as bioactive molecules that cross epithelial barriers and exert systemic immunomodulatory effects (72, 101). This crosstalk could explain how distal microbiota, such as gut microbiota, influence responses to cutaneous tumors.

Dietary interventions and prebiotic or probiotic strategies are being explored as low-cost, non-invasive means to reshape the microbiota and potentiate anticancer therapies. Specific diets rich in fiber, omega-3 fatty acids, and polyphenols have been shown to favorably modulate gut microbial composition and may synergize with ICIs or delay melanoma progression (80). However, translating these findings into clinical practice will require large-scale, controlled trials and real-time microbiome monitoring tools to guide patient stratification and therapeutic personalization.

In conclusion, the microbial dimension of melanoma is emerging as a pivotal axis in both tumor biology and therapeutic responsiveness. Far from being a passive passenger, the microbiome actively shapes local and systemic immune landscapes, influences tumor evolution, and modulates the efficacy of frontline treatments. Future directions should prioritize the integration of microbial biomarkers into clinical stratification frameworks, the development of standardized microbiota-targeted interventions, and the incorporation of microbial therapeutics as adjuncts to existing immuno-oncology protocols. Unlocking the full therapeutic potential of the host-microbe crosstalk may not only transform our approach to melanoma but also establish new paradigms in precision cancer medicine.

## References

[1] Leiter U Meier F Schittek B Garbe C. The natural course of cutaneous melanoma. *J Surg Oncol.* (2004) 86:172–8. 10.1002/jso.20079 15221923

[2] Bray F Laversanne M Sung H Ferlay J Siegel R Soerjomataram I Global cancer statistics 2022: GLOBOCAN estimates of incidence and mortality worldwide for 36 cancers in 185 countries. *CA Cancer J Clin.* (2024) 74:229–63. 10.3322/caac.21834 38572751

[3] Liu C Liu X Hu L Li X Xin H Zhu S. Global, regional, and national burden of cutaneous malignant melanoma from 1990 to 2021 and prediction to 2045. *Front Oncol.* (2024) 14:1512942. 10.3389/fonc.2024.1512942 39777336 PMC11703817

[4] Mekadim C Skalnikova H Cizkova J Cizkova V Palanova A Horak V Dysbiosis of skin microbiome and gut microbiome in melanoma progression. *BMC Microbiol.* (2022) 22:63. 10.1186/s12866-022-02458-5 35216552 PMC8881828

[5] Zhu Y Liu W Wang M Wang X Wang S. Causal roles of skin microbiota in skin cancers suggested by genetic study. *Front Microbiol.* (2024) 15:1426807. 10.3389/fmicb.2024.1426807 39161599 PMC11330880

[6] Chen Y Bousbaine D Veinbachs A Atabakhsh K Dimas A Yu V Engineered skin bacteria induce antitumor T cell responses against melanoma. *Science.* (2023) 380:203–10. 10.1126/science.abp9563 37053311 PMC12356174

[7] Liu X Chen Y Zhang S Dong L. Gut microbiota-mediated immunomodulation in tumor. *J Exp Clin Cancer Res.* (2021) 40:221. 10.1186/s13046-021-01983-x 34217349 PMC8254267

[8] Lee K Thomas A Bolte L Björk J de Ruijter L Armanini F Cross-cohort gut microbiome associations with immune checkpoint inhibitor response in advanced melanoma. *Nat Med.* (2022) 28:535–44. 10.1038/s41591-022-01695-5 35228751 PMC8938272

[9] Xie M Li X Lau H Yu J. The gut microbiota in cancer immunity and immunotherapy. *Cell Mol Immunol.* (2025) 22:1012–31. 10.1038/s41423-025-01326-2 40770084 PMC12398524

[10] Lu Y Yuan X Wang M He Z Li H Wang J Gut microbiota influence immunotherapy responses: mechanisms and therapeutic strategies. *J Hematol Oncol.* (2022) 15:47. 10.1186/s13045-022-01273-9 35488243 PMC9052532

[11] Davar D Dzutsev A McCulloch J Rodrigues R Chauvin J Morrison R Fecal microbiota transplant overcomes resistance to anti-PD-1 therapy in melanoma patients. *Science.* (2021) 371:595–602. 10.1126/science.abf3363 33542131 PMC8097968

[12] Irfan M Delgado R Frias-Lopez J. The oral microbiome and cancer. *Front Immunol.* (2020) 11:591088. 10.3389/fimmu.2020.591088 33193429 PMC7645040

[13] Liu W Yang X Zhou Y Huang Z Huang J. Gut microbiota in melanoma: effects and pathogeneses. *Microbiol Res.* (2025) 296:128144. 10.1016/j.micres.2025.128144 40120565

[14] Byrd A Belkaid Y Segre J. The human skin microbiome. *Nat Rev Microbiol.* (2018) 16:143–55. 10.1038/nrmicro.2017.157 29332945

[15] Grice E Segre J. The skin microbiome. *Nat Rev Microbiol.* (2011) 9:244–53. 21407241 10.1038/nrmicro2537 PMC3535073

[16] Severn M Horswill A. Staphylococcus epidermidis and its dual lifestyle in skin health and infection. *Nat Rev Microbiol.* (2023) 21:97–111. 10.1038/s41579-022-00780-3 36042296 PMC9903335

[17] Chen Y Fischbach M Belkaid Y. Skin microbiota-host interactions. *Nature.* (2018) 553:427–36. 10.1038/nature25177 29364286 PMC6075667

[18] Li X Zhang S Guo G Han J Yu J. Gut microbiome in modulating immune checkpoint inhibitors. *EBioMedicine.* (2022) 82:104163. 10.1016/j.ebiom.2022.104163 35841869 PMC9297075

[19] Zhang H Xie Y Cao F Song X. Gut microbiota-derived fatty acid and sterol metabolites: biotransformation and immunomodulatory functions. *Gut Microbes.* (2024) 16:2382336. 10.1080/19490976.2024.2382336 39046079 PMC11271093

[20] Ranjbar R Vahdati S Tavakoli S Khodaie R Behboudi H. Immunomodulatory roles of microbiota-derived short-chain fatty acids in bacterial infections. *Biomed Pharmacother.* (2021) 141:111817. 10.1016/j.biopha.2021.111817 34126349

[21] Yousefi Y Baines K Maleki Vareki S. Microbiome bacterial influencers of host immunity and response to immunotherapy. *Cell Rep Med.* (2024) 5:101487. 10.1016/j.xcrm.2024.101487 38547865 PMC11031383

[22] Gunjur A Shao Y Rozday T Klein O Mu A Haak B A gut microbial signature for combination immune checkpoint blockade across cancer types. *Nat Med.* (2024) 30:797–809. 10.1038/s41591-024-02823-z 38429524 PMC10957475

[23] Tan J McKenzie C Potamitis M Thorburn A Mackay C Macia L. The role of short-chain fatty acids in health and disease. *Adv Immunol.* (2014) 121:91–119. 10.1016/B978-0-12-800100-4.00003-9 24388214

[24] Kim M Kang S Park J Yanagisawa M Kim C. Short-chain fatty acids activate GPR41 and GPR43 on intestinal epithelial cells to promote inflammatory responses in mice. *Gastroenterology.* (2013) 145:396–406.e1. 10.1053/j.gastro.2013.04.056 23665276

[25] Singh N Gurav A Sivaprakasam S Brady E Padia R Shi H Activation of Gpr109a, receptor for niacin and the commensal metabolite butyrate, suppresses colonic inflammation and carcinogenesis. *Immunity.* (2014) 40:128–39. 10.1016/j.immuni.2013.12.007 24412617 PMC4305274

[26] Davie J. Inhibition of histone deacetylase activity by butyrate. *J Nutr.* (2003) 133:2485S–93S. 10.1093/jn/133.7.2485S 12840228

[27] Park J Kim M Kang S Jannasch A Cooper B Patterson J Short-chain fatty acids induce both effector and regulatory T cells by suppression of histone deacetylases and regulation of the mTOR-S6K pathway. *Mucosal Immunol.* (2015) 8:80–93. 10.1038/mi.2014.44 24917457 PMC4263689

[28] Luu M Pautz S Kohl V Singh R Romero R Lucas S The short-chain fatty acid pentanoate suppresses autoimmunity by modulating the metabolic-epigenetic crosstalk in lymphocytes. *Nat Commun.* (2019) 10:760. 10.1038/s41467-019-08711-2 30770822 PMC6377655

[29] Xiao X Hu X Yao J Cao W Zou Z Wang L The role of short-chain fatty acids in inflammatory skin diseases. *Front Microbiol.* (2022) 13:1083432. 10.3389/fmicb.2022.1083432 36817115 PMC9932284

[30] Hezaveh K Shinde R Klötgen A Halaby M Lamorte S Ciudad M Tryptophan-derived microbial metabolites activate the aryl hydrocarbon receptor in tumor-associated macrophages to suppress anti-tumor immunity. *Immunity.* (2022) 55:324–340.e8. 10.1016/j.immuni.2022.01.006 35139353 PMC8888129

[31] Xue C Li G Zheng Q Gu X Shi Q Su Y Tryptophan metabolism in health and disease. *Cell Metab.* (2023) 35:1304–26. 10.1016/j.cmet.2023.06.004 37352864

[32] Ginefra P Hope H Chiang Y Nutten S Blum S Coukos G Urolithin-A promotes CD8+ T Cell-mediated cancer immunosurveillance via FOXO1 activation. *Cancer Res Commun.* (2024) 4:1189–98. 10.1158/2767-9764.CRC-24-0022 38626334 PMC11067828

[33] Karumuru V Dhasmana A Mamidi N Chauhan S Yallapu M. Unveiling the potential of Urolithin A in cancer therapy: mechanistic insights to future perspectives of nanomedicine. *Nanotheranostics.* (2025) 9:121–43. 10.7150/ntno.110966 40568370 PMC12188533

[34] Kouhzad M Götz F Navidifar T Taki E Ghamari M Mohammadzadeh R Carcinogenic and anticancer activities of microbiota-derived secondary bile acids. *Front Oncol.* (2025) 15:1514872. 10.3389/fonc.2025.1514872 39944822 PMC11813773

[35] Huang D Wang Y Li B Wu L Xie W Zhou X Association between periodontal disease and systemic diseases: a cross-sectional analysis of current evidence. *Mil Med Res.* (2024) 11:74. 10.1186/s40779-024-00583-y 39633497 PMC11616297

[36] Routy B Jackson T Mählmann L Baumgartner C Blaser M Byrd A Melanoma and microbiota: current understanding and future directions. *Cancer Cell.* (2024) 42:16–34. 10.1016/j.ccell.2023.12.003 38157864 PMC11096984

[37] Valencia J Erwin-Cohen R Clavijo P Allen C Sanford M Day C Myeloid-derived suppressive cell expansion promotes melanoma growth and autoimmunity by inhibiting CD40/IL27 regulation in macrophages. *Cancer Res.* (2021) 81:5977–90. 10.1158/0008-5472.CAN-21-1148 34642183 PMC8639618

[38] Jimenez-Sanchez M Celiberto L Yang H Sham H Vallance B. The gut-skin axis: a bi-directional, microbiota-driven relationship with therapeutic potential. *Gut Microbes.* (2025) 17:2473524. 10.1080/19490976.2025.2473524 40050613 PMC11901370

[39] Spencer C McQuade J Gopalakrishnan V McCulloch J Vetizou M Cogdill A Dietary fiber and probiotics influence the gut microbiome and melanoma immunotherapy response. *Science.* (2021) 374:1632–40. 10.1126/science.aaz7015 34941392 PMC8970537

[40] Gopalakrishnan V Spencer C Nezi L Reuben A Andrews M Karpinets T Gut microbiome modulates response to anti-PD-1 immunotherapy in melanoma patients. *Science.* (2018) 359:97–103. 10.1126/science.aan4236 29097493 PMC5827966

[41] Patra V Byrne S Wolf P. The skin microbiome: is it affected by uv-induced immune suppression? *Front Microbiol.* (2016) 7:1235. 10.3389/fmicb.2016.01235 27559331 PMC4979252

[42] Derosa L Hellmann M Spaziano M Halpenny D Fidelle M Rizvi H Negative association of antibiotics on clinical activity of immune checkpoint inhibitors in patients with advanced renal cell and non-small-cell lung cancer. *Ann Oncol.* (2018) 29:1437–44. 10.1093/annonc/mdy103 29617710 PMC6354674

[43] Sun J Song S Liu J Chen F Li X Wu G. Gut microbiota as a new target for anticancer therapy: from mechanism to means of regulation. *NPJ Biofilms Microb.* (2025) 11:43. 10.1038/s41522-025-00678-x 40069181 PMC11897378

[44] Hoxhaj G Manning B. The PI3K-AKT network at the interface of oncogenic signalling and cancer metabolism. *Nat Rev Cancer.* (2020) 20:74–88. 10.1038/s41568-019-0216-7 31686003 PMC7314312

[45] Jiang M Zhang K Zhang Z Zeng X Huang Z Qin P Pi3k/akt/mtor axis in cancer: from pathogenesis to treatment. *MedComm.* (2025) 6:e70295. 10.1002/mco2.70295 40740483 PMC12308072

[46] Steelman L Chappell W Abrams S Kempf R Long J Laidler P Roles of the Raf/MEK/ERK and PI3K/PTEN/Akt/mTOR pathways in controlling growth and sensitivity to therapy-implications for cancer and aging. *Aging.* (2011) 3:192–222. 10.18632/aging.100296 21422497 PMC3091517

[47] Rossi T Vergara D Fanini F Maffia M Bravaccini S Pirini F. Microbiota-derived metabolites in tumor progression and metastasis. *Int J Mol Sci.* (2020) 21:5786. 10.3390/ijms21165786 32806665 PMC7460823

[48] Yang Q Wang B Zheng Q Li H Meng X Zhou F A review of gut microbiota-derived metabolites in tumor progression and cancer therapy. *Adv Sci.* (2023) 10:e2207366. 10.1002/advs.202207366 36951547 PMC10214247

[49] Monti M Benerini Gatta L Bugatti M Pezzali I Picinoli S Manfredi M Novel cellular systems unveil mucosal melanoma initiating cells and a role for PI3K/Akt/mTOR pathway in mucosal melanoma fitness. *J Transl Med.* (2024) 22:35. 10.1186/s12967-023-04784-2 38191367 PMC10775657

[50] Li Z Xiong W Liang Z Wang J Zeng Z Kołat D Critical role of the gut microbiota in immune responses and cancer immunotherapy. *J Hematol Oncol.* (2024) 17:33. 10.1186/s13045-024-01541-w 38745196 PMC11094969

[51] Lunjani N Ahearn-Ford S Dube F Hlela C O’Mahony L. Mechanisms of microbe-immune system dialogue within the skin. *Genes Immun.* (2021) 22:276–88. 10.1038/s41435-021-00133-9 33993202 PMC8497273

[52] Zhang S Lv K Liu Z Zhao R Li F. Fatty acid metabolism of immune cells: a new target of tumour immunotherapy. *Cell Death Discov.* (2024) 10:39. 10.1038/s41420-024-01807-9 38245525 PMC10799907

[53] Dokoshi T Chen Y Cavagnero K Rahman G Hakim D Brinton S Dermal injury drives a skin to gut axis that disrupts the intestinal microbiome and intestinal immune homeostasis in mice. *Nat Commun.* (2024) 15:3009. 10.1038/s41467-024-47072-3 38589392 PMC11001995

[54] Belkaid Y Segre J. Dialogue between skin microbiota and immunity. *Science.* (2014) 346:954–9. 10.1126/science.1260144 25414304

[55] Johnston E Heras B Kufer T Kaparakis-Liaskos M. Detection of bacterial membrane vesicles by NOD-like receptors. *Int J Mol Sci.* (2021) 22:1005. 10.3390/ijms22031005 33498269 PMC7863931

[56] Wicherska-Pawłowska K Wróbel T Rybka J. Toll-like receptors (TLRs), NOD-like receptors (NLRs), and RIG-I-like receptors (RLRs) in innate immunity. TLRs, NLRs, and RLRs ligands as immunotherapeutic agents for hematopoietic diseases. *Int J Mol Sci.* (2021) 22:3397. 10.3390/ijms222413397 34948194 PMC8704656

[57] Mrázek J Mekadim C Kučerová P Švejstil R Salmonová H Vlasáková J Melanoma-related changes in skin microbiome. *Folia Microbiol.* (2019) 64:435–42. 10.1007/s12223-018-00670-3 30554379

[58] Li Z Peres A Damian A Madrenas J. Immunomodulation and Disease Tolerance to Staphylococcus aureus. *Pathogens.* (2015) 4:793–815. 10.3390/pathogens4040793 26580658 PMC4693165

[59] Jin Z Song Y He L. A review of skin immune processes in acne. *Front Immunol.* (2023) 14:1324930. 10.3389/fimmu.2023.1324930 38193084 PMC10773853

[60] Flacher V Bouschbacher M Verronèse E Massacrier C Sisirak V Berthier-Vergnes O Human Langerhans cells express a specific TLR profile and differentially respond to viruses and Gram-positive bacteria. *J Immunol.* (2006) 177:7959–67. 10.4049/jimmunol.177.11.7959 17114468

[61] Tong Y Lu G Wang Z Hao S Zhang G Sun H. Tubeimuside I improves the efficacy of a therapeutic *Fusobacterium nucleatum* dendritic cell-based vaccine against colorectal cancer. *Front Immunol.* (2023) 14:1154818. 10.3389/fimmu.2023.1154818 37207216 PMC10189021

[62] Ren C Zhang Q de Haan B Zhang H Faas M de Vos P. Identification of TLR2/TLR6 signalling lactic acid bacteria for supporting immune regulation. *Sci Rep.* (2016) 6:34561. 10.1038/srep34561 27708357 PMC5052581

[63] Jiao Y Wu L Huntington N Zhang X. Crosstalk between gut microbiota and innate immunity and its implication in autoimmune diseases. *Front Immunol.* (2020) 11:282. 10.3389/fimmu.2020.00282 32153586 PMC7047319

[64] Perl M Fante M Herfeld K Scherer J Poeck H Thiele Orberg E. Microbiota-derived metabolites: key modulators of cancer immunotherapies. *Med.* (2025) 6:100773. 10.1016/j.medj.2025.100773 40695291

[65] Yuan C He Y Xie K Feng L Gao S Cai L. Review of microbiota gut brain axis and innate immunity in inflammatory and infective diseases. *Front Cell Infect Microbiol.* (2023) 13:1282431. 10.3389/fcimb.2023.1282431 37868345 PMC10585369

[66] Li C Jiang P Wei S Xu X Wang J. Regulatory T cells in tumor microenvironment: new mechanisms, potential therapeutic strategies and future prospects. *Mol Cancer.* (2020) 19:116. 10.1186/s12943-020-01234-1 32680511 PMC7367382

[67] Huang L Guo Y Liu S Wang H Zhu J Ou L Targeting regulatory T cells for immunotherapy in melanoma. *Mol Biomed.* (2021) 2:11. 10.1186/s43556-021-00038-z 34806028 PMC8591697

[68] He Y Huang J Li Q Xia W Zhang C Liu Z Gut microbiota and tumor immune escape: a new perspective for improving tumor immunotherapy. *Cancers.* (2022) 14:5317. 10.3390/cancers14215317 36358736 PMC9656981

[69] Xie H Xi X Lei T Liu H Xia Z. CD8+ T cell exhaustion in the tumor microenvironment of breast cancer. *Front Immunol.* (2024) 15:1507283. 10.3389/fimmu.2024.1507283 39717767 PMC11663851

[70] Gros A Robbins P Yao X Li Y Turcotte S Tran E PD-1 identifies the patient-specific CD8+ tumor-reactive repertoire infiltrating human tumors. *J Clin Invest.* (2014) 124:2246–59. 10.1172/JCI73639 24667641 PMC4001555

[71] Kau A Ahern P Griffin N Goodman A Gordon J. Human nutrition, the gut microbiome and the immune system. *Nature.* (2011) 474:327–36. 10.1038/nature10213 21677749 PMC3298082

[72] Scharschmidt T Segre J. Skin microbiome and dermatologic disorders. *J Clin Invest.* (2025) 135:4315. 10.1172/JCI184315 39895627 PMC11785926

[73] Arafat Hossain MA. comprehensive review of immune checkpoint inhibitors for cancer treatment. *Int Immunopharmacol.* (2024) 143:113365. 10.1016/j.intimp.2024.113365 39447408

[74] Botticelli A Zizzari I Mazzuca F Ascierto P Putignani L Marchetti L Cross-talk between microbiota and immune fitness to steer and control response to anti PD-1/PDL-1 treatment. *Oncotarget.* (2017) 8:8890–9. 10.18632/oncotarget.12985 27806346 PMC5352451

[75] Peng K Li Y Yang Q Yu P Zeng T Lin C The therapeutic promise of probiotic *Bacteroides fragilis* (BF839) in cancer immunotherapy. *Front Microbiol.* (2025) 16:1523754. 10.3389/fmicb.2025.1523754 40231233 PMC11995047

[76] Vétizou M Pitt J Daillère R Lepage P Waldschmitt N Flament C Anticancer immunotherapy by CTLA-4 blockade relies on the gut microbiota. *Science.* (2015) 350:1079–84. 10.1126/science.aad1329 26541610 PMC4721659

[77] Routy B Le Chatelier E Derosa L Duong C Alou M Daillère R Gut microbiome influences efficacy of PD-1-based immunotherapy against epithelial tumors. *Science.* (2018) 359:91–7. 10.1126/science.aan3706 29097494

[78] Lam K Araya R Huang A Chen Q Di Modica M Rodrigues R Microbiota triggers STING-type I IFN-dependent monocyte reprogramming of the tumor microenvironment. *Cell.* (2021) 184:5338–5356.e21. 10.1016/j.cell.2021.09.019 34624222 PMC8650838

[79] Makaranka S Scutt F Frixou M Wensley K Sharma R Greenhowe J. The gut microbiome and melanoma: a review. *Exp Dermatol.* (2022) 31:1292–301. 10.1111/exd.14639 35793428

[80] Kumar P Brazel D DeRogatis J Valerin J Whiteson K Chow W The cure from within? A review of the microbiome and diet in melanoma. *Cancer Metastasis Rev.* (2022) 41:261–80. 10.1007/s10555-022-10029-3 35474500 PMC9042647

[81] Łyko M Maj J Jankowska-Konsur A. The role of the gut microbiome in non-hodgkin lymphoma (NHL): a focus on diffuse large B-cell lymphoma, follicular lymphoma, cutaneous T-cell lymphoma, and NK/T-cell lymphoma. *Cancers.* (2025) 17:1709. 10.3390/cancers17101709 40427206 PMC12110234

[82] Baruch E Youngster I Ben-Betzalel G Ortenberg R Lahat A Katz L Fecal microbiota transplant promotes response in immunotherapy-refractory melanoma patients. *Science.* (2021) 371:602–9. 10.1126/science.abb5920 33303685

[83] Lim S Shklovskaya E Lee J Pedersen B Stewart A Ming Z The molecular and functional landscape of resistance to immune checkpoint blockade in melanoma. *Nat Commun.* (2023) 14:1516. 10.1038/s41467-023-36979-y 36934113 PMC10024679

[84] Liu J Liu C Yue J. Radiotherapy and the gut microbiome: facts and fiction. *Radiat Oncol.* (2021) 16:9. 10.1186/s13014-020-01735-9 33436010 PMC7805150

[85] Taitz J Tan J Ni D Potier-Villette C Grau G Nanan R Antibiotic-mediated dysbiosis leads to activation of inflammatory pathways. *Front Immunol.* (2024) 15:1493991. 10.3389/fimmu.2024.1493991 39850904 PMC11754057

[86] Adlakha Y Chhabra R. The human microbiome: redefining cancer pathogenesis and therapy. *Cancer Cell Int.* (2025) 25:165. 10.1186/s12935-025-03787-x 40296128 PMC12039184

[87] Gulliver E Young R Chonwerawong M D’Adamo G Thomason T Widdop J Review article: the future of microbiome-based therapeutics. *Aliment Pharmacol Ther.* (2022) 56:192–208. 10.1111/apt.17049 35611465 PMC9322325

[88] Hitch T Hall L Walsh S Leventhal G Slack E de Wouters T Microbiome-based interventions to modulate gut ecology and the immune system. *Mucosal Immunol.* (2022) 15:1095–113. 10.1038/s41385-022-00564-1 36180583 PMC9705255

[89] Cho Y Han K Xu J Moon J. Novel strategies for modulating the gut microbiome for cancer therapy. *Adv Drug Deliv Rev.* (2024) 210:115332. 10.1016/j.addr.2024.115332 38759702 PMC11268941

[90] Zitvogel L Derosa L Kroemer G. Modulation of cancer immunotherapy by dietary fibers and over-the-counter probiotics. *Cell Metab.* (2022) 34:350–2. 10.1016/j.cmet.2022.02.004 35235771

[91] Bolte L Lee K Björk J Leeming E Campmans-Kuijpers M de Haan J Association of a mediterranean diet with outcomes for patients treated with immune checkpoint blockade for advanced melanoma. *JAMA Oncol.* (2023) 9:705–9. 10.1001/jamaoncol.2022.7753 36795408 PMC9936383

[92] Merrick B Allen L Masirah M Zain N Forbes B Shawcross DL Regulation, risk and safety of faecal microbiota transplant. *Infect Prev Pract.* (2020) 2:100069. 10.1016/j.infpip.2020.100069 34316559 PMC7280140

[93] Yadegar A Bar-Yoseph H Monaghan T Pakpour S Severino A Kuijper E Fecal microbiota transplantation: current challenges and future landscapes. *Clin Microbiol Rev.* (2024) 37:e0006022. 10.1128/cmr.00060-22 38717124 PMC11325845

[94] Grossi P Wolfe C Peghin M. Non-standard risk donors and risk of donor-derived infections: from evaluation to therapeutic management. *Transpl Int.* (2024) 37:12803. 10.3389/ti.2024.12803 39416809 PMC11479921

[95] Haugh A Salama A Johnson D. Advanced melanoma: resistance mechanisms to current therapies. *Hematol Oncol Clin North Am.* (2021) 35:111–28. 10.1016/j.hoc.2020.09.005 33759769 PMC7991196

[96] Glitza I Seo Y Spencer C Wortman J Burton E Alayli F Randomized placebo-controlled, biomarker-stratified phase IB microbiome modulation in melanoma: impact of antibiotic preconditioning on microbiome and immunity. *Cancer Discov.* (2024) 14:1161–75. 10.1158/2159-8290.CD-24-0066 38588588 PMC11215408

[97] Guo W Wang H Li C. Signal pathways of melanoma and targeted therapy. *Signal Transduct Target Ther.* (2021) 6:424. 10.1038/s41392-021-00827-6 34924562 PMC8685279

[98] Bautista J Lopez-Cortes A. Oncogenic viruses rewire the epigenome in human cancer. *Front Cell Infect Microbiol.* (2025) 15:1617198. 10.3389/fcimb.2025.1617198 40557320 PMC12185521

[99] Bautista J Ojeda-Mosquera S Ordóñez-Lozada D López-Cortés A. Peripheral clocks and systemic zeitgeber interactions: from molecular mechanisms to circadian precision medicine. *Front Endocrinol.* (2025) 16:1606242. 10.3389/fendo.2025.1606242 40510487 PMC12158691

[100] Pérez-Villa A Echeverría-Garcés G Ramos-Medina M Prathap L Martínez-López M Ramírez-Sánchez D Integrated multi-omics analysis reveals the molecular interplay between circadian clocks and cancer pathogenesis. *Sci Rep.* (2023) 13:14198. 10.1038/s41598-023-39401-1 37648722 PMC10469199

[101] Bautista J Ávila-Coello D Hidalgo-Tinoco C Bueno-Miño J López-Cortés A. Unraveling the gut-brain-immune interplay in herpes simplex virus-associated neurodegeneration. *J Med Virol.* (2025) 97:e70504. 10.1002/jmv.70504 40704582

